# Causes, Characteristics, and Risk Factors of Medical Damage Liability Disputes: A 13-Year Retrospective Analysis of 1761 Litigation Cases in Chongqing, China

**DOI:** 10.3390/healthcare14142047

**Published:** 2026-07-08

**Authors:** Xue Zhang, Chuan Pu

**Affiliations:** 1 School of Public Health, Chongqing Medical University, Chongqing 400016, China; 18580073779@163.com; 2 Research Center for Medicine and Social Development, Chongqing Medical University, Chongqing 400016, China; 3 Collaborative Innovation Center for Early Warning of Health-Related Major Social Risks, Chongqing Medical University Sub Center, Chongqing 400016, China

**Keywords:** medical damage liability disputes, adjudication documents, doctor–patient relationship, risk prevention and control, Chongqing

## Abstract

**Objective:** To investigate the occurrence trends, distribution characteristics, and contributing factors of medical damage liability disputes in Chongqing from 2012 to 2024, thereby providing data support for risk prevention and control in regional medical institutions and for health policy formulation. **Methods:** A retrospective study design was employed. Adjudication documents pertaining to medical damage liability disputes in Chongqing, spanning from January 2012 to December 2024, were retrieved from the China Judgments Online database. A total of 1761 eligible cases were selected based on standardized criteria. Statistical analysis was conducted on core case variables. **Results:** The number of cases exhibited a fluctuating upward trend from 2012 to 2024, peaking in 2019 (276 cases) before subsequently declining. The average actual compensation amount demonstrated a steady increase, reaching its highest level in 2023 (259,200 ± 53,900 CNY). Deficiencies in diagnostic and therapeutic procedures constituted the predominant cause of disputes (50.37%). Surgical departments (46.05%) and orthopedics departments (22.60%) were identified as high-risk specialties. Tertiary public hospitals accounted for the highest proportion of disputes (48.89%) and the highest average actual compensation (216,100 ± 10,600 CNY). Regarding the apportionment of liability, secondary liability attributed to the medical provider was the most frequent (29.59%). Patient death was the most common adverse outcome (37.14%). The highest average compensation amount was associated with persistent vegetative state outcomes (777,500 ± 157,200 CNY). Statistically significant differences in actual compensation amounts were observed across all outcome categories (all *p* < 0.05). **Conclusions:** The situation concerning medical damage disputes in Chongqing remains severe. The etiologies of disputes are predominantly attributable to medical technical factors—particularly deficiencies in diagnostic and therapeutic procedures—and failure to fulfill a duty of care, both of which are associated with elevated compensation amounts. Given that this study employed a retrospective descriptive design, the aforementioned findings reflect associations among variables rather than causal relationships. Based on the identified risk hierarchy, it is recommended that priority be given to strengthening perioperative safety verification and re-certification of critical procedural skills in high-incidence departments, such as surgery and obstetrics and gynecology, enhancing informed consent and medical record quality management, and advancing the tiered healthcare delivery system, with a view to reducing medical risks and mitigating doctor–patient conflicts.

## 1. Introduction

Currently, the persistently high incidence of medical disputes and the strained, complex nature of doctor–patient relationships in China have emerged as focal issues of significant societal concern [1]. Medical damage liability disputes represent the primary legal mechanism through which patients and healthcare providers seek judicial resolution of their conflicts. Such disputes refer to civil liability controversies arising from personal injuries sustained by patients due to the fault of medical institutions or their staff during diagnostic and therapeutic activities [2]. In recent years, with the growing health demands of the populace, widespread enhancement of legal awareness, and the successive implementation of laws and regulations such as the Civil Code, the occurrence of medical damage liability disputes has shown an increasing trend [3]. The rapid escalation in the total caseload not only imposes substantial economic and psychological burdens on both parties involved but also, to a certain extent, disrupts normal medical order and adversely affects the construction of a harmonious society [4].

Chongqing Municipality, as the sole municipality directly under the central government in Western China, possesses a large population base, and the distribution of medical and health resources exhibits marked regional disparities [5]. The core metropolitan area hosts a concentration of tertiary Grade A public hospitals with robust medical service capabilities, whereas the level of primary healthcare services in outlying suburban counties and districts remains comparatively underdeveloped. At present, research specifically addressing medical damage liability disputes in Chongqing remains relatively scarce. Existing studies generally lack large-sample, long-term systematic analyses, and empirical research based on adjudication document data is even more deficient. Consequently, current evidence is insufficient to provide reliable data support for risk prevention and control in local medical institutions and the formulation of health policies. As a quintessential urban configuration characterized by the coexistence of a large metropolis, vast rural areas, extensive mountainous regions, and a major reservoir area, Chongqing simultaneously embodies the high-quality medical resource clusters typical of China’s developed eastern regions and the primary care deficits characteristic of the less developed western regions. Its dual urban–rural structure and the degree of regional developmental imbalance exceed those observed in most other provinces nationwide. This distinctive socioeconomic and healthcare delivery landscape renders Chongqing a setting where medical disputes may exhibit both a concentration toward high-tier institutions due to resource siphoning effects in the core metropolitan area, and divergent etiological and compensation patterns relative to national averages attributable to the weak capacity of primary care services. Accordingly, an empirical analysis using Chongqing as the study sample not only supplements prior research, which has predominantly focused on eastern provinces or nationally pooled samples, but also facilitates the examination of the applicability of existing conclusions in the context of a western municipality directly under the central government.

In summary, prior research on medical damage liability disputes in China has largely concentrated on single-center, short-duration, or national macro-level investigations. A systematic characterization of a single western municipality over an extended period utilizing a comprehensive corpus of adjudication documents has been lacking. Furthermore, few studies have integrated dispute etiology, involved clinical departments, institutional tier, degree of liability, and damage outcomes within a unified analytical framework for cross-interpretation. This study draws upon 13 years of regional adjudication document data spanning the complete judicial practice period following the implementation of the Tort Liability Law, and constructs an integrated analytical framework structured around etiology—liability—damage—compensation. It is intended to provide reliable, localized, empirical evidence to facilitate the precise identification of risk nodes by medical institutions in western regions, as well as the formulation of regionally targeted prevention and control policies by health administrative authorities. This also constitutes the principal innovation distinguishing the present study from prior retrospective investigations.

Therefore, this study utilizes litigation cases of medical damage liability disputes in Chongqing, publicly available within the China Judgments Online database, as the research sample. The study period spans from January 2012 to December 2024. Following rigorous screening, a total of 1761 eligible cases were ultimately included for analysis. A retrospective analysis was conducted to statistically examine key indicators, including the year of case occurrence, actual compensation amounts, patient damage outcomes, degree of medical liability, involved clinical departments, and hospital tiers. This study systematically explores the overall trends and intrinsic causes of medical damage disputes in Chongqing, with the objective of providing feasible countermeasures and practical references for medical institutions to scientifically manage medical risks and mitigate the incidence of medical disputes.

## 2. Materials and Methods

### 2.1. Data Source and Study Subjects

This study utilized the China Judgments Online database as its data source. This platform, officially established in 2013 by the Supreme People’s Court of the People’s Republic of China, constitutes the nation’s largest and most authoritative government-operated platform for the public disclosure of judicial documents, systematically archiving adjudication documents from courts at all levels nationwide for cases that have been concluded [6]. All documents accessible via this website are publicly released only after court review, thereby ensuring high data reliability and robust traceability. It has consequently become a vital data source for large-scale retrospective studies in the fields of legal studies and medical damage disputes. The specific retrieval parameters for this study were set as follows: case cause of action restricted to “medical damage liability dispute”; document type selected as “judgment”; geographical region limited to “Chongqing Municipality”; and judgment date spanning from 1 January 2012 to 31 December 2024. This specific timeframe was selected as the study window for the following reasons. First, although the China Judgments Online platform was officially launched in 2013, it subsequently underwent retroactive supplementation and incorporation of certain adjudication documents from 2012. Employing 2012 as the temporal starting point permits the maximum inclusion of early-stage electronically disclosed judicial adjudication documents within the country, thereby expanding sample coverage. Second, this time interval comprehensively encompasses the judicial practice cycle following the implementation of the Tort Liability Law of the People’s Republic of China in 2010, while simultaneously incorporating critical policy and public health event phases, including the deepening healthcare system reforms of 2015 and the COVID-19 pandemic spanning 2020–2023. This coverage enables an objective reflection of the potential impact of various external environmental changes on medical damage liability dispute litigation in the Chongqing region, systematically reveals the long-term evolution characteristics of such cases, and effectively enhances the temporal stability of study findings and the validity of their extrapolation. A preliminary search yielded a total of 2204 judgment documents. It should be noted that the China Judgments Online database exhibits a retroactive uploading phenomenon for early adjudication documents, particularly those from the 2012–2014 period. The volume of publicly accessible cases may be influenced by factors such as document disclosure policies and the pace of judicial publication, and thus does not necessarily correspond precisely to the actual incidence of disputes. To mitigate the potential selection bias arising from this issue, this study adopted a cautious strategy in interpreting annual trends: on the one hand, all available cases were included to maximize the coverage of early documents; on the other hand, in both the Section 3 and Section 4, the low case counts observed in the early years were treated as likely attributable to incomplete reporting or disclosure rather than being used as a basis for definitive inferences regarding dispute incidence rates. A detailed explanation is provided in Section 4.5.

The inclusion criteria were as follows: (1) documents publicly accessible via the China Judgments Online database; (2) clear documentation of patient injury outcomes; (3) case cause of action or adjudication result directly pertinent to medical damage liability disputes; (4) explicit record of compensation amounts; (5) availability of effective court judgments or forensic expert opinions; and (6) adjudication rendered by courts within the jurisdiction of Chongqing Municipality.

The exclusion criteria were as follows: (1) duplicate case entries; (2) cases concluded via mediation, withdrawal of lawsuit, or dismissal of action; (3) cases with missing critical information, including cause of dispute, degree of medical liability, damage outcomes, or compensation amounts; (4) cases where the cause of action was not classified as a medical damage dispute; and (5) documents classified as court rulings rather than judgments.

Based on the aforementioned inclusion and exclusion criteria, the preliminary search yielded a total of 2204 documents from the China Judgments Online database. After applying the exclusion criteria, the following were removed: (1) duplicate case entries (n = 182); (2) documents not publicly accessible (n = 55); (3) cases dismissed by the court (n = 75); and (4) cases with missing critical information, including cause of dispute, degree of medical liability, damage outcomes, or compensation amounts (n = 131). Ultimately, a total of 1761 valid judgments were included in the statistical analysis.

### 2.2. Study Methods

This study employed a retrospective study design, involving the selection of 1761 adjudicated medical dispute cases finalized between January 2012 and December 2024 as the study population. Based on the core content of the adjudication documents, the researchers designed data extraction forms, subsequently completing data entry and conducting statistical analysis. The analytical dimensions primarily encompassed: (1) general case characteristics; (2) distribution of dispute etiologies; (3) distribution of involved clinical departments; (4) distribution of involved medical institution types; (5) apportionment of medical liability; and (6) patient damage outcomes.

Data extraction was performed using a structured data extraction form independently designed by the research team based on the core elements of the adjudication documents. This form was organized around the six analytical dimensions described above, with explicit classification criteria established for each dimension: (1) general case characteristics (continuous/count variables such as adjudication year and actual compensation amount); (2) dispute etiology (categorized according to a “primary injury-oriented causation” principle into nine primary categories and their respective secondary subcategories: deficiencies in diagnostic and therapeutic procedures, deficiencies in communication and informed consent management, failure to fulfill duty of care, diagnostic deficiencies, deficiencies in nursing service management, medical product issues, deficiencies in medical documentation management, hospital management deficiencies, and improper medication use or adverse drug reactions; see Supplementary Table S1 and its notes for details); (3) involved clinical departments (classified using a hybrid system of “administrative categories + high-frequency/high-risk subspecialties” based on the standard diagnostic and therapeutic disciplines issued by the National Health Commission; see Supplementary Table S2 and its notes for details); (4) type of involved medical institution (four categories: primary public, secondary public, tertiary public, and private institutions); (5) degree of medical liability (classified as no liability, slight, secondary, equal, primary, or full liability in accordance with the Guidelines for Medical Damage Appraisal issued by the Ministry of Justice and the conclusions of forensic expert opinions); and (6) patient damage outcomes (graded by disability level and death, among other outcomes).

To ensure data quality and reproducibility, a rigorous data extraction and verification protocol was established. First, duplicate case identification was performed through joint deduplication using three fields—“case number + patient information + medical institution information”—supplemented by manual review to ensure that the same incident was not included more than once. Second, records with incomplete or ambiguous information were handled as follows: records missing critical fields such as age, sex, or compensation amount were excluded; missing values for non-critical variables were imputed using multiple imputation (m = 5), and the robustness of the results was verified through sensitivity analyses. Third, a three-stage data cleaning and verification process was implemented: (a) terminology standardization—a synonym standardization dictionary was developed to uniformly map different phrasings describing the same type of negligence in the original text (e.g., “improper operation” and “surgical error”) to the designated classification codes; (b) logical cross-validation incorporating case metadata was conducted to verify the consistency between the sum of itemized compensation components and the total amount, with records exhibiting logical inconsistencies or missing critical data flagged for review or exclusion; and (c) dual independent manual verification was performed by two researchers who compared the extracted data against the original adjudication documents on an item-by-item basis, with any discrepancies or disagreements resolved through discussion or consultation with a third senior expert.

With regard to the coding of dispute etiologies and liability categories, all coding was independently completed by two researchers with clinical medical backgrounds, and inter-coder reliability was assessed using Cohen’s Kappa. For items with insufficient agreement (Kappa < 0.8), a third senior expert was introduced to arbitrate and ensure coding consistency. When a single adjudication document involved multiple points of contention, this study applied the primary contributing factor principle for coding—that is, the factor identified by the forensic expert opinion or court judgment as the most direct and most heavily weighted cause of the injury outcome was selected as the primary etiology for that case, to avoid statistical bias arising from multiple coding. It should be noted that all compensation amounts across the 13-year study period were recorded at their current/nominal values as stated in the adjudication documents, without adjustment for inflation (price indices). Therefore, longitudinal comparisons of compensation amounts across years should be interpreted descriptively. A detailed explanation is provided in Section 4.5.

### 2.3. Statistical Analysis

All statistical analyses were performed using SPSS 27.0 software, with a two-sided significance level of *p* < 0.05. Categorical data were described as frequencies and proportions (%). Compensation amounts, as continuous monetary data, were described as mean ± standard error ($\bar{x}$ ± s), with 95% confidence intervals (95% CI) of the mean. Prior to analysis, the distributional characteristics of compensation amounts were assessed: normality was evaluated using Q-Q plots, skewness, and kurtosis values, while homogeneity of variance was assessed using Levene’s test. Given that compensation amounts exhibited a right-skewed (positively skewed) distribution with heterogeneity of variance in most subgroups, Welch’s F-test (or the Brown–Forsythe test) was applied to correct the main effect in the presence of variance heterogeneity. For post hoc pairwise comparisons, the LSD-t test was used when equal variances were assumed, and the Games-Howell test when equal variances were not assumed; Tukey’s HSD method was applied to generate homogeneous subsets for identifying group hierarchies.

Effect sizes were reported as partial eta squared (ηp^2^) to quantify the degree to which each factor explained the variance in compensation amounts, thereby avoiding over-reliance on *p*-values alone. Given the skewed distribution of monetary data, non-parametric Kruskal–Wallis H tests were conducted as sensitivity analyses, and their conclusions were consistent with the parametric tests, further supporting the robustness of the results. Since this study performed multiple group comparisons across several dimensions (etiology, department, institutional tier, degree of liability, and damage outcome), to control for Type I error inflation, the aforementioned post hoc correction methods robust to variance heterogeneity were uniformly applied for within-group pairwise comparisons, and results were interpreted with careful consideration of effect sizes and confidence intervals to assess practical significance. For subgroups with extremely small sample sizes (e.g., endocrinology, n = 2; infectious diseases, n = 3), the estimates exhibited considerable variability with wide confidence intervals; therefore, the relevant results are only descriptively presented and should not be used for definitive inference.

### 2.4. Ethics Statement

This study involved secondary analysis of publicly available, de-identified adjudication document data obtained from the China Judgments Online database. According to the policy of the authors’ institution, additional ethical approval was not required for this type of study.

## 3. Results

### 3.1. General Characteristics of Medical Damage Dispute Cases

A statistically significant difference was observed in the average actual compensation amounts among medical damage dispute cases across different adjudication years (F = 1.943, *p* = 0.026 < 0.05). The effect size, as measured by partial eta squared (ηp^2^), was 0.013, indicating that adjudication year accounted for only a small proportion of the variance in compensation amounts. Given the presence of variance heterogeneity, the main effect was corrected using Welch’s F-test, and Games-Howell post hoc tests were employed for pairwise comparisons. The annual distribution of cases and the corresponding average compensation amounts are detailed in Table 1. Regarding case volume, the incidence of medical disputes demonstrated an overall upward trend from 2012 to 2024. A notable surge in the number of disputes occurred after 2016, with the caseload in 2017 approximately tripling compared to the preceding year. The number of cases peaked in 2019 at 276 cases, subsequently exhibiting a gradual decline thereafter. Concerning adjudication outcomes, the actual compensation amounts awarded by court judgments showed an overall increasing trend from 2012 to 2024. The average compensation amount surpassed 200,000 CNY in 2023, reaching 259,200 CNY, which represented a relatively elevated level during the study period. It should be noted that the gradual decline in case volume after 2019 may not solely reflect a genuine reduction in the actual incidence of disputes, but may also be attributable to the combined effects of adjustments to adjudication document disclosure policies, changes in the pace of judicial publication, and case filing restrictions imposed during the COVID-19 pandemic. Similarly, the low case counts observed during the 2012–2014 period may have been influenced by retroactive uploading of adjudication documents and incomplete online disclosure. Accordingly, this study presents annual trends in case volume only as descriptive observations and does not draw causal inferences regarding changes in the incidence of disputes based on these trends.

### 3.2. Distribution of Etiologies of Medical Damage Dispute Cases

A statistically significant difference was observed in the average actual compensation amounts among cases attributed to different dispute etiologies (F = 2.069, *p* < 0.001). When compared at the level of primary etiological category, the between-group differences were also statistically significant (primary category: F = 4.574, *p* < 0.001). The effect sizes, measured as partial eta squared (ηp^2^), were 0.034 for secondary categories and 0.020 for primary categories, respectively, suggesting that dispute etiology as a whole accounted for only a limited proportion of the variance in compensation amounts. Given the heterogeneity of variance across etiology groups and the considerable imbalance in sample sizes—which ranged from as few as 3 to as many as 350 cases—the main effect remained statistically significant following Welch correction and Brown–Forsythe robustness tests; Games-Howell post hoc procedures were applied for pairwise comparisons. The distribution of etiologies and the corresponding average compensation amounts are detailed in Supplementary Table S1. Based on a comprehensive assessment incorporating both case proportion and average compensation amount, the nine categories of dispute etiologies were stratified into three distinct tiers. Tier I comprised deficiencies in diagnostic and therapeutic procedures, which accounted for the highest proportion of individual cases (50.37%) and the highest average actual compensation (221,200 CNY), thereby representing the predominant etiology of medical damage disputes. Tier II included failure to fulfill duty of care and diagnostic deficiencies. These two categories exhibited comparable case proportions (approximately 14.0% each) and similar average compensation amounts (approximately 180,000 CNY), characterizing them as etiologies with moderate incidence and moderate compensation levels. Tier III encompassed the remaining etiologies, each accounting for less than 10% of total cases. Among these, medical product issues constituted the lowest proportion (1.65%) and the lowest average compensation (96,200 CNY), categorizing it as a low-incidence, low-compensation etiology.

### 3.3. Distribution of Involved Clinical Departments in Medical Damage Dispute Cases

A statistically significant difference was observed in the average actual compensation amounts among medical damage dispute cases across different clinical departments (F = 2.145, *p* < 0.001). The effect sizes, as measured by partial eta squared, were ηp^2^ = 0.014 for primary categories and ηp^2^ = 0.039 for secondary categories, respectively. Given the presence of variance heterogeneity, Games-Howell post hoc pairwise comparisons were employed. Tukey HSD homogeneous subset analysis revealed that primary departmental categories could be classified into three homogeneous subsets, indicating significant stratification among broad administrative categories; however, at the secondary subspecialty level, all groups were assigned to a single subset, suggesting that between-group differences in compensation amounts did not reach statistical significance at the granularity of individual subspecialties. It should be noted that certain subspecialties had extremely small sample sizes (e.g., endocrinology, n = 2; infectious diseases, n = 3); the corresponding mean estimates exhibit considerable variability with wide confidence intervals, and the relevant results are presented descriptively only. The distribution of involved departments and the corresponding average compensation amounts are detailed in Supplementary Table S2. Among the seven primary departmental categories, surgical departments accounted for the highest proportion of disputes (46.05%), thereby identified as high-risk settings for medical disputes. Within this category, the orthopedics department represented 22.60% of cases, exhibiting the highest risk of dispute occurrence among all secondary departments. Conversely, among primary departments, general practice accounted for the lowest proportion of disputes (4.20%). At the secondary department level, the endocrinology department was associated with the fewest number of cases, totaling only two cases. Adjudication outcomes revealed that, among primary departmental categories, the pediatrics department recorded the highest average compensation amount (260,700 CNY). At the secondary department level, the otorhinolaryngology (ENT) department registered the highest average compensation (304,800 CNY). The lowest compensation amounts were associated with general practice (110,800 CNY) at the primary level and the infectious diseases department (20,000 CNY) at the secondary level.

### 3.4. Distribution of Hospital Types in Medical Damage Dispute Cases

A statistically significant difference was observed in the average actual compensation amounts among medical damage dispute cases across different hospital types (F = 9.198, *p* < 0.001). The effect size, as measured by partial eta squared, was ηp^2^ = 0.015. Levene’s test indicated highly significant variance heterogeneity (*p* < 0.001); therefore, Games-Howell post hoc pairwise comparisons were conducted. The results revealed that the compensation amounts awarded in cases involving private medical institutions were significantly lower than those involving secondary public hospitals and tertiary public hospitals (*p* < 0.001), whereas no statistically significant differences were observed among primary, secondary, and tertiary public hospitals, which were classified into the same homogeneous subset. This finding suggests that institutional nature (public vs. private) exerts a more pronounced influence on compensation amounts than institutional tier alone. The distribution of cases and the corresponding average actual compensation amounts by hospital type are detailed in Table 2 (total sample, n = 1761). Both the number of dispute cases and the average actual compensation amounts associated with secondary public hospitals and tertiary public hospitals exceeded those of private medical institutions. Tertiary public hospitals were identified as high-incidence settings for medical damage disputes, accounting for 861 cases—the highest caseload among all institutional categories—with an average compensation amount of 216,100 CNY, which also ranked highest among all institution types. Primary public hospitals exhibited the lowest incidence of medical disputes, constituting only 8.4% of total cases, representing less than half the caseload of private medical institutions. However, the average actual compensation amount in primary public hospitals was 185,500 CNY, which remained higher than that observed in private medical institutions (130,700 CNY).

### 3.5. Distribution of Liability Apportionment in Medical Damage Dispute Cases

A statistically significant difference was observed in the average actual compensation amounts among medical damage dispute cases involving different degrees of medical liability (F = 40.222, *p* < 0.001). The effect size, as measured by partial eta squared, was ηp^2^ = 0.121, indicating that the degree of liability possessed relatively strong explanatory power for the variance in compensation amounts. Given the substantial disparities in standard deviations across groups, Welch’s F-test served as the primary basis for inference regarding the main effect (Welch F = 206.327, *p* < 0.001), and Tukey’s HSD method was employed for post hoc multiple comparisons. It should be noted that the 24 cases solely involving compensation for moral damages—a category that does not fall within the fault participation grading framework—were excluded from the analysis of liability degree; accordingly, the analysis presented in this section pertains to the remaining 1737 cases. The distribution of liability apportionment and the corresponding average actual compensation amounts are detailed in Table 3. The results indicated that cases in which medical institutions bore secondary liability constituted the highest proportion, accounting for 29.99% of all cases, whereas cases involving a finding of no liability accounted for the lowest proportion, at 6.16%. Regarding average actual compensation amounts, cases in which medical institutions bore primary liability resulted in the highest compensation awards (313,800 CNY), while cases with a finding of no liability yielded the lowest average actual compensation (5300 CNY). Overall, compensation amounts exhibited a stepwise upward trend corresponding to increasing degrees of liability.

### 3.6. Distribution of Damage Outcomes in Medical Damage Dispute Cases

A statistically significant difference was observed in the average actual compensation amounts among medical damage dispute cases involving different damage outcomes (F = 47.003, *p* < 0.001). The effect size, as measured by partial eta squared, was ηp^2^ = 0.288, making damage outcome the strongest predictor of compensation amount variance identified in this study. Given the marked disparities in sample sizes across groups and the substantial fluctuation in standard deviations, Welch’s F-test (Welch F = 59.191, *p* < 0.001) was employed in place of conventional ANOVA for the main effect. Tukey HSD homogeneous subset analysis revealed that the 16 damage outcome categories could be classified into nine hierarchical tiers, with compensation amounts exhibiting a pronounced stepwise escalation trend corresponding to increasing injury severity: the lowest compensation tier encompassed minor injury and cases not reaching disability assessment thresholds, while the highest compensation tier included Grade 1 disability and persistent vegetative state. The distribution of cases by damage outcome and the corresponding average actual compensation amounts are detailed in Table 4. Grade 1 disability is the most severe, while Grade 10 disability is the mildest. The smaller the disability grade number, the higher the degree of disability and the more severe the physical function impairment. Among the 1761 cases analyzed, patient death constituted the most frequent outcome, accounting for 654 cases (37.14%), with an average actual compensation amount of 215,900 CNY. A persistent vegetative state represented the least frequent outcome, comprising only 20 cases (1.14%); however, this outcome category recorded the highest average actual compensation amount at 777,500 CNY. Cases involving minor patient injury corresponded to the lowest average actual compensation amount.

## 4. Discussion

This study adopted an analytical framework structured around the logic of etiology—liability—damage—compensation to systematically identify the primary drivers and high-risk nodes of medical damage liability disputes. Dispute etiologies were categorized into two overarching dimensions: medical technical negligence and hospital administrative deficiencies. Case distribution characteristics were then cross-analyzed with institutional tier, clinical department category, degree of liability, and damage outcomes. This framework aims to provide medical institutions with actionable entry points for targeted risk prevention and control. It should be noted at the outset that this study is a retrospective descriptive analysis. All relationships between the various etiologies and compensation amounts or damage outcomes discussed below should be understood as statistical associations rather than empirically established causal pathways. The relevant interpretations are intended to provide testable hypotheses for future research.

### 4.1. Analysis of the Current Status and Governance of Medical Damage Disputes

In China, medical damage liability dispute cases have persisted at elevated levels over an extended period [7]. Nationwide, such cases approached approximately 19,000 between 2019 and 2020. Since 2021, influenced by adjustments to adjudication document disclosure policies and the promotion of diversified mediation mechanisms, the number of publicly available cases has declined. Nevertheless, the total volume of litigation remains substantial. In national adjudication practice, the proportion of cases in which medical providers are found liable for compensation is high, with failure to fulfill a duty of care accounting for approximately 30% of adverse judgments against medical institutions. Against this backdrop, this study focuses on 1761 cases from Chongqing Municipality, analyzing their etiological characteristics and evolutionary patterns.

Regarding the fundamental landscape of medical damage liability disputes, the number of cases exhibited an overall upward trend, punctuated by two notable periods of decline. The first decline occurred between 2015 and 2016, during which the number of cases dropped significantly from 167 in the preceding year to 95. This downward trend is closely associated with the clarification of relevant legal boundaries and the intensified crackdown on medical-related criminal offenses. The promulgation of the Opinions on Lawfully Punishing Medical-Related Crimes to Maintain Normal Medical Order in 2014, followed by the formal inclusion of “medical disturbance” (Yi Nao) within the scope of criminal law through the 2015 amendment to the Criminal Law, established a legal foundation for the prevention and control of doctor–patient disputes [8]. The second decline, observed between 2020 and 2021, is presumably linked to the COVID-19 pandemic’s prevention and control measures. On one hand, influenced by epidemic containment protocols, courts may have implemented stricter controls over case filing volumes [9]. On the other hand, patients may have opted to postpone litigation due to increased procedural complexity and heightened difficulty in navigating the case filing process. With specific reference to Chongqing, China, beyond the national-level pandemic prevention and control policies, as a western municipality directly under the central government with substantial population mobility, the city implemented region-specific, tiered, and targeted containment measures during the pandemic, along with adjustments to the operational order of medical institutions and phased restrictions on in-person case filing and court proceedings. These factors may have further delayed the entry of disputes into the litigation process, thereby amplifying the decline in publicly available case volume observed during the 2020–2021 period. It should be emphasized that the aforementioned annual fluctuations in case counts may also have been influenced by adjudication document disclosure policies and the pace of judicial publication. Consequently, these trends reflect only the volume of cases that entered litigation and were publicly disclosed, and should not necessarily be equated with actual changes in the incidence of disputes. The relevant interpretations should be approached with caution.

From the perspective of contributory fault, the analysis of the study sample indicates that cases involving a finding of no liability on the part of the medical institution accounted for only 6.08%. Conversely, in 93.92% of adjudicated cases, the court determined that the medical institution had committed diagnostic or therapeutic errors and should bear corresponding liability, underscoring a relatively high overall rate of compensable fault attributable to medical providers. Regarding the apportionment of liability, cases in which medical institutions bore secondary liability constituted the largest proportion, followed by those involving primary liability. This distribution pattern is broadly consistent with adjudication trends observed in national medical damage liability dispute cases over the past five years [10]. In judicial practice, courts typically rely on statutes such as the Regulations on Handling Medical Accidents and the Interpretation of the Supreme People’s Court on Several Issues Concerning the Application of Law in the Trial of Medical Damage Liability Dispute Cases, alongside established judicial precedents. The core basis for adjudication rests upon forensic expert opinions in medical damage assessments, which are employed to comprehensively evaluate the causal relationship between diagnostic or therapeutic conduct and the resulting injury, as well as the degree of contributory fault, ultimately determining the scope of compensation liability borne by the medical institution [11].

Collectively, the study findings underscore the persistently challenging landscape of medical damage disputes. In recent years, legislative instruments including the Regulations on the Prevention and Handling of Medical Disputes, the Administrative Measures for Complaints in Medical Institutions, and the Civil Code have been successively enacted. These regulations delineate immediate procedures for handling medical disputes, emphasize resolving doctor–patient conflicts through flexible legal approaches, and reinforce the role of mediation as a primary resolution channel. They provide diversified pathways for medical institutions to prevent disputes at their source, maintain medical order, combat medical-related criminal activities, and facilitate effective doctor–patient communication. Research indicates that following the implementation of these regulations, the proportion of disputes resolved through medical mediation has gradually increased. Nonetheless, the number of disputes proceeding to litigation remains substantial, suggesting that shifting the threshold of dispute resolution further upstream remains a formidable and ongoing endeavor [12].

### 4.2. Technical Etiologies of Medical Damage Disputes and Strategies for Enhancing Service Quality

This study identified diagnostic deficiencies and deficiencies in diagnostic and therapeutic procedures as two principal etiologies precipitating medical damage disputes, a finding consistent with prior studies [13,14]. Among the 1761 cases analyzed, 887 cases involved deficiencies in diagnostic and therapeutic procedures, with an average actual compensation amount reaching 220,000 CNY, establishing this category as the predominant factor in medical fault. The most prevalent subcategories within this domain were improper treatment processes and insufficient intraoperative precision. Medical fault cases arising from diagnostic deficiencies totaled 247, primarily manifesting as missed diagnoses, incomplete diagnostic coverage, and misdiagnosis. Notably, cases involving misdiagnosis registered an average actual compensation amount as high as 230,000 CNY. These findings indicate considerable scope for improvement in the quality of contemporary healthcare services. From a technical standpoint, limited or uneven professional competencies among certain healthcare personnel may impede their ability to comprehensively and accurately assess patient conditions, predisposing them to diagnostic errors [15]. Furthermore, discord between patient expectations and actual therapeutic outcomes—particularly when such outcomes lack adequate explanation—can readily engender doctor–patient tensions [16]. Additionally, modern medicine inherently possesses distinct limitations. The diagnosis and management of many diseases present inherent difficulties, and in the context of complex or refractory conditions, misdiagnosis, missed diagnosis, or suboptimal treatment plans cannot be entirely eliminated [17]. Such occurrences may result in patient harm, thereby provoking dissatisfaction among patients and their families, and ultimately culminating in medical disputes.

Consequently, medical institutions may adopt a tripartite approach to enhance healthcare quality and mitigate the incidence of medical disputes. First, institutions should strengthen the professional training of healthcare personnel by routinely organizing specialized knowledge acquisition, various forms of continuing education, and conducting skill-based operational training and competency assessments to promote standardized healthcare delivery. Specifically, targeted simulation-based training modules can be designed to address the high-frequency error types identified in this study—namely, insufficient intraoperative precision and improper treatment processes—and a system of periodic retraining and re-certification for key operational skills can be established to ensure that performance quality in core diagnostic and therapeutic procedures is continuously maintained at acceptable standards. Second, efforts should be directed toward facilitating knowledge renewal among healthcare personnel. Given the rapid advancement of medical technology, institutions committed to a strategy of talent-driven development should provide opportunities for external training and academic exchange, enabling healthcare personnel to acquire proficiency in the latest medical technologies and concepts through systematic learning and practical application [10]. It is recommended that external advanced training be moderately linked to departmental performance evaluations, and that returning trainees be required to submit a technology transfer report or conduct an intra-departmental training lecture, thereby facilitating effective knowledge dissemination at the departmental level and preventing individual learning gains from failing to translate into team competencies. Third, institutions should actively adopt and deploy novel medical technologies and advanced equipment, leveraging technological innovation to establish high-quality clinical platforms and drive substantial improvements in healthcare service delivery. Concurrently, it is imperative to ensure that relevant operators possess appropriate qualifications and competencies to prevent disputes arising from improper equipment handling or procedural execution [18]. Prior to the introduction of new equipment or technologies, a mandatory learning curve assessment period and a qualification-based credential system should be instituted. This ensures that operators complete a prescribed number of supervised procedures and pass a competency assessment before being authorized to perform clinical work independently.

### 4.3. Deficiencies in Hospital Administration and Preventive Countermeasures

This study categorized the etiologies of medical disputes into nine distinct classifications. Among these, two categories pertained to medical technical negligence, while medical product issues and medication-related adverse reactions accounted for additional categories. The remaining five categories were attributable to hospital administrative deficiencies, collectively encompassing 541 cases. Within administrative disputes, the three most prevalent subcategories were failure to fulfill a duty of care, deficiencies in communication and informed consent management, and deficiencies in medical documentation management. Among communication and disclosure issues, failure to fulfill disclosure obligations was most prominent and frequently served as a critical basis for judicial determination of compensation ratios. The three categories associated with the highest compensation amounts were non-compliance with practice qualifications, failure to fulfill nursing responsibilities, and falsification of medical records. From a management systems perspective, inadequacies or suboptimal execution of policies governing medical resource allocation, clinical workflows, medical record management, and nosocomial infection control can directly compromise healthcare quality and safety, thereby precipitating adverse medical safety events [19,20,21,22]. While medical technology possesses objective boundaries, hospital administration must not harbor ambiguous domains. Administrative loopholes constitute a significant contributing factor to the high incidence of medical disputes [13]. With the progressive implementation of novel medical technologies and policies promoting multi-site physician practice, relevant regulations have lagged, hospital management mechanisms remain underdeveloped, and the delineation of liability remains ambiguous, collectively exacerbating latent risks for doctor–patient conflicts.

Patient safety constitutes the core objective of hospital management [23]. Medical institutions should adopt a patient-centered approach, systematically identify administrative deficiencies, and implement closed-loop management across the entire diagnostic and therapeutic continuum. First, institutions must rigorously fulfill their disclosure and explanation obligations, fully respect patient autonomy, and standardize informed consent procedures [24]. Refining informed-consent-related institutional frameworks serves as a cornerstone for establishing trust between clinicians and patients. A standardized informed consent form template library can be developed, with procedure-specific consent documents formulated for common invasive procedures, high-risk treatment plans, and alternative treatment options within each department. The standardized execution of informed consent procedures should be incorporated into the medical record quality audit process to ensure that the consent process is thoroughly documented and verifiable. Second, institutions should integrate standardized medical record management into internal quality control assessments. Properly maintained medical records safeguard legitimate patient rights and interests while simultaneously reinforcing awareness of lawful practice and providing legal protection for healthcare personnel [25]. It is recommended that a time-sensitive reminder and quality control feedback mechanism for medical record documentation be established, along with a departmental medical record quality liaison system. Monthly sampling inspections of archived medical records should be conducted, with quality control results publicly disseminated and linked to quarterly departmental quality performance assessments. Finally, institutions should elaborate upon the duty of care and nursing safety protocols, and strengthen the continuing education system. Regular training and assessment focused on high-risk clinical scenarios should be implemented, alongside the establishment of safety assurance mechanisms commensurate with patient risk profiles, to mitigate the occurrence of related adverse events [26,27]. In the domain of nursing safety, a nursing procedure risk checklist can be developed targeting the high-frequency adverse events identified in this study—such as infusion/injection-related issues and infections resulting from improper nursing procedures—and a dual-person verification system for high-risk nursing procedures can be implemented to systematically reduce the risk of errors in nursing care [28].

### 4.4. Workload Burden in Tertiary Public Hospitals and Inadequate Oversight of High-Risk Departments

Regarding hospital tier distribution, the findings of this study demonstrate that the incidence of medical damage disputes in tertiary public hospitals significantly exceeds that observed in other categories of medical institutions [29,30]. This finding aligns with large-scale data reports from the past five years, which consistently identify tertiary general hospitals as the primary institutional settings for medical damage liability dispute litigation [19].

Influenced by demographic transitions, evolving disease spectra, and the siphoning effect of healthcare-seeking behavior, tertiary public hospitals remain the predominant venue for initial patient consultations. The substantial utilization of premium healthcare resources by patients with common or chronic conditions results in chronic operational overload for these institutions, consequently elevating the risk of medical damage disputes. Moreover, most tertiary public hospitals concurrently bear teaching responsibilities, compelling clinical physicians to allocate considerable attention to research endeavors and the management of complex cases, thereby impeding their capacity for meticulous patient management. Furthermore, some interns or visiting trainees possess insufficient clinical experience, rendering them prone to procedural errors that may directly cause patient harm and further amplify the risk of medical litigation. In response, continued advancement of the tiered healthcare delivery system is warranted to invigorate primary care service capacity and enhance patients’ willingness to seek care at grassroots institutions. Leveraging integrated healthcare consortia and regional medical centers, the adoption of unified management models should be promoted to bolster primary care diagnostic and therapeutic capabilities, thereby guiding patients with common or frequently occurring conditions toward locally accessible care and fostering an orderly, tiered healthcare landscape. In terms of concrete measures, unified referral criteria and green-channel protocols can be established within healthcare consortia, alongside the institutionalization of a routine mechanism whereby physicians from higher-tier hospitals rotate through primary care facilities for scheduled clinical consultations and on-site mentorship. This approach would substantively elevate the quality of primary care delivery, thereby strengthening patient trust in initially presenting at grassroots institutions.

Concerning clinical department distribution, this study’s findings indicate that surgical departments accounted for the highest number of litigation cases, with orthopedics identified as a high-risk secondary specialty. This result is broadly consistent with conclusions drawn in prior research [31]. Surgical disciplines are characterized by a high density of invasive procedures, complex patient conditions, and elevated surgical risks, rendering lapses in perioperative management more likely and thereby precipitating medical disputes [32]. Orthopedic patients disproportionately comprise older adults who frequently present with multiple comorbid conditions, thereby substantially augmenting diagnostic and therapeutic risk [33]. Concurrently, orthopedics manages a considerable volume of acute traumatic injuries and polytrauma cases, where missed diagnoses, misdiagnosis, or treatment delays are more prone to occur, culminating in disputes [34]. Collectively, these factors contribute to the designation of surgical and orthopedics departments as high-incidence settings for medical disputes. To address this, a proactive early warning risk mechanism and an active prevention and control framework should be established in key clinical departments to enhance the capacity for medical risk mitigation in high-risk specialties. On one hand, technological solutions should be harnessed to optimize standardized workflows in critical diagnostic and therapeutic processes, thereby reducing adverse events attributable to human operational variance. On the other hand, leveraging big data models can facilitate closed-loop management of clinical practice behaviors, enabling early warning for high-risk cases and, through comprehensive quality traceability across the care continuum, diminishing the probability of high-severity adverse events. In practice, a mandatory electronic pre-operative safety checklist system can be piloted in orthopedic and surgical departments, designed to ensure that verification steps cannot be skipped or omitted. Concurrently, for emergency trauma patients, a multidisciplinary joint assessment pathway can be established, stipulating that imaging examinations, laboratory tests, and specialist consultations be completed within a defined time window following admission, thereby systematically reducing the risk of missed diagnoses and treatment delays. Based on the risk hierarchy identified in this study, it is recommended that tertiary public hospitals implement the aforementioned measures in a stepwise manner, following the principle of prioritizing high-incidence departments and high-compensation procedures first. The first priority should target the department with the highest dispute volume—surgery—and its high-risk subspecialty, orthopedics, where mandatory electronic pre-operative safety checklists and re-certification systems for critical procedural skills should be implemented in the perioperative setting without delay. The second priority should focus on diagnostic and therapeutic procedures associated with the highest compensation damage outcomes (e.g., severe disability, persistent vegetative state, and death), strengthening closed-loop management and early warning systems for high-risk cases. The third priority should then extend to other high-compensation departments such as obstetrics and pediatrics, as well as to high-frequency administrative deficiencies (e.g., failure to fulfill disclosure obligations and falsification of medical records). This approach ensures that limited quality improvement resources are first directed toward the most risk-concentrated nodes, thereby enhancing the marginal benefit of interventions.

### 4.5. Limitations

This study is subject to several limitations. First, it employed a retrospective analysis of adjudication documents, which is inherently a descriptive and associational study design; therefore, causal inferences regarding the relationships between dispute etiologies and case outcomes cannot be established. The associations identified herein—such as high-risk departments and high-frequency etiologies—may inform risk prevention efforts but should not be interpreted as causal pathways. Second, the study sample was limited to Chongqing Municipality, and the extrapolation of findings to other regions of China or to international settings requires caution. Future studies incorporating multi-center data or employing prospective designs are warranted to further validate these findings. Third, at the level of statistical inference, this study relied primarily on univariate comparisons. The factors examined—institutional tier, clinical department, degree of liability, damage outcome, and others—may be interrelated, and residual confounding may exist. As adjudication documents do not comprehensively record clinical details such as the severity of patients’ underlying diseases, comorbidities, or socioeconomic status, these variables could not be controlled for; consequently, the between-group differences observed may be driven in part by unmeasured variables. Fourth, the coding of dispute etiologies and liability categories depended on the manual interpretation of unstructured adjudication documents. Although this was addressed through dual independent coding and consistency testing, the classification inevitably carries a degree of subjectivity and uncertainty. Fifth, certain subgroups had extremely small sample sizes (e.g., endocrinology, n = 2; infectious diseases, n = 3; drug allergy events, n = 3), yielding mean estimates with substantial variability and wide confidence intervals. The statistical stability of these estimates is limited, and the corresponding results should be regarded as descriptive references only rather than a basis for definitive inference. Sixth, annual fluctuations in case volume may be simultaneously influenced by multiple factors, including the actual incidence of disputes, adjudication document disclosure policies, the pace of judicial publication, and case filing restrictions; the interpretation of temporal trends should therefore remain cautious. Seventh, this study did not adjust compensation amounts for inflation, and cross-year comparisons were performed using nominal values. This may, to some extent, overestimate the true magnitude of compensation growth in later years. Finally, given the descriptive orientation of this study, the association signals identified in high-risk departments such as surgery and orthopedics should be regarded as hypothesis-generating for subsequent research. Future studies may progressively transition from descriptive analyses to prospective cohort studies, and ultimately to interventional trials targeting specific high-risk nodes (e.g., evaluating the actual impact of mandatory electronic pre-operative safety checklists and critical procedural re-certification on dispute incidence and compensation outcomes), thereby elevating the level of evidence and strengthening the guidance value for clinical practice.

## 5. Conclusions

Based on 1761 adjudication documents concerning medical damage liability disputes in Chongqing, China, spanning the period from 2012 to 2024, this study systematically characterized the temporal distribution, etiological composition, involved clinical departments, institutional tiers, degrees of liability, and damage outcome profiles of these disputes, as well as their associations with compensation amounts. The findings indicate that medical damage disputes in Chongqing exhibited an overall fluctuating upward trend. Deficiencies in diagnostic and therapeutic procedures constituted the predominant etiology of disputes; surgical departments were identified as high-incidence settings, and tertiary public hospitals as high-incidence institutions. The degree of liability and damage outcome emerged as the factors most strongly associated with compensation amounts, which displayed a stepwise escalation corresponding to increasing severity in both dimensions. It should be emphasized that the foregoing conclusions derive from a retrospective descriptive design and reflect statistical associations among variables rather than causal relationships.

Accordingly, this study proposes targeted prevention and control recommendations to be implemented in a stepwise manner according to risk hierarchy: (1) mandatory electronic pre-operative safety checklists and re-certification of critical procedural skills should be prioritized in high-incidence departments such as surgery and obstetrics and gynecology, with initial implementation in the perioperative setting; (2) for diagnostic and therapeutic procedures associated with high-compensation outcomes such as severe disability, persistent vegetative state, and death, closed-loop management and early warning systems for high-risk cases should be strengthened; (3) informed consent procedures and medical record quality management should be standardized, and source governance of high-frequency administrative deficiencies—such as failure to fulfill disclosure obligations and falsification of medical records—should be reinforced; and (4) the tiered healthcare delivery system and the development of integrated healthcare consortia should be further advanced to alleviate the operational overload of tertiary public hospitals. These measures may provide localized empirical references for medical institutions in western China to implement precision-oriented medical risk prevention, and for health administrative authorities to formulate regionally tailored policies. Future research should build upon these findings and progressively advance from descriptive analyses toward prospective and interventional studies to verify the actual effectiveness of the aforementioned measures.

## Figures and Tables

**Table 1:** General Characteristics of Medical Damage Dispute Cases in China from 2012 to 2024.

Adjudication Year	Number of Cases (n)	Proportion (%)	Average Actual Compensation ($\bar{x}$ ± s)/10,000 CNY	95% CI (LL, UL)
2012	7	0.40%	14.33 ± 6.74	(−2.1774, 30.8289)
2013	30	1.70%	14.84 ± 4.18	(6.2839, 23.4021)
2014	176	9.99%	14.55 ± 1.77	(11.0667, 18.0386)
2015	167	9.48%	15.51 ± 1.59	(12.3754, 18.6412)
2016	95	5.39%	19.19 ± 3.38	(12.4854, 25.8902)
2017	271	15.39%	18.61 ± 1.64	(15.3853, 21.8349)
2018	236	13.40%	17.83 ± 1.51	(14.8588, 20.7931)
2019	276	15.67%	23.11 ± 1.82	(19.526, 26.6953)
2020	193	10.96%	21.43 ± 1.89	(17.7076, 25.1457)
2021	151	8.57%	21.69 ± 2.20	(17.3554, 26.0344)
2022	63	3.58%	22.63 ± 2.64	(17.3451, 27.9111)
2023	45	2.56%	25.92 ± 5.39	(15.0497, 36.7849)
2024	51	2.90%	19.59 ± 3.38	(12.8001, 26.3701)

**Table 2:** Distribution of Hospital Types in Medical Damage Dispute Cases in China from 2012 to 2024.

Hospital Types	Number of Cases (n)	Proportion (%)	Average Actual Compensation ($\bar{x}$ ± s)/10,000 CNY	95% CI (LL, UL)
Primary Public Hospital	148	8.40	18.55 ± 1.92	(14.7591, 22.3351)
Secondary Public Hospital	398	22.60	20.54 ± 1.11	(18.3585, 22.7164)
Tertiary Public Hospital	861	48.89	21.61 ± 1.06	(19.5227, 23.6948)
Private Medical Institution	354	20.10	13.07 ± 0.90	(11.3073, 14.8301)

Note: Percentages may not sum to exactly 100% due to rounding.

**Table 3:** Distribution of Liability Apportionment in Medical Damage Dispute Cases in China from 2012 to 2024.

Degree of Liability	Number of Cases (n)	Proportion (%)	Average Actual Compensation ($\bar{x}$ ± s)/10,000 CNY	95% CI (LL, UL)
Slight Liability	257	14.80%	7.99 ± 0.43	(7.1466, 8.8304)
Secondary Liability	521	29.99%	16.79 ± 0.74	(15.3388, 18.251)
Equal Liability	290	16.70%	23.86 ± 1.38	(21.1442, 26.5681)
Primary Liability	426	24.53%	31.38 ± 1.9	(27.6453, 35.1093)
Full Liability	136	7.83%	21.5 ± 2.42	(16.7103, 26.2919)
No Liability	107	6.16%	0.53 ± 0.14	(0.2465, 0.8189)

Note: Cases solely involving compensation for moral damages (N = 24), which do not fall within the fault participation grading framework, were excluded from this table. The analysis of liability degree was conducted on the remaining 1737 cases.

**Table 4:** Distribution of Damage Outcomes in Medical Damage Dispute Cases in China from 2012 to 2024.

Damage Outcome	Number of Cases (n)	Proportion (%)	Average Actual Compensation ($\bar{x}$ ± s)/10,000 CNY	95% CI (LL, UL)
Minor Injury	61	3.46	2.34 ± 0.35	(1.6427, 3.0334)
Disability Assessment Not Reached	71	4.03	4.81 ± 0.55	(3.7104, 5.9195)
Disability Grade Unspecified	182	10.34	6.52 ± 0.71	(5.1259, 7.9211)
Grade 10 Disability	191	10.85	8.50 ± 0.54	(7.4467, 9.5575)
Grade 9 Disability	169	9.60	11.97 ± 0.79	(10.4029, 13.5313)
Grade 8 Disability	95	5.39	16.21 ± 1.20	(13.8258, 18.5913)
Grade 7 Disability	72	4.09	25.84 ± 2.53	(20.8046, 30.8829)
Grade 6 Disability	41	2.33	22.67 ± 3.45	(15.6994, 29.646)
Grade 5 Disability	40	2.27	36.37 ± 6.53	(23.1653, 49.5772)
Grade 4 Disability	34	1.93	24.50 ± 3.87	(16.6278, 32.3692)
Grade 3 Disability	36	2.04	48.23 ± 9.74	(28.4528, 68.0072)
Grade 2 Disability	21	1.19	52.71 ± 12.23	(27.2041, 78.2064)
Grade 1 Disability	50	2.84	67.68 ± 8.23	(51.1339, 84.2245)
Persistent Vegetative State	20	1.14	77.75 ± 15.72	(44.8396, 110.6614)
Fetal Death	24	1.36	12.93 ± 3.96	(4.7448, 21.116)
Death	654	37.14	21.59 ± 0.73	(20.1528, 23.0334)

## Data Availability

The data presented in this study are available from China Judgments Online at https://wenshu.court.gov.cn/.

## Supplementary Materials

The following supporting information can be downloaded at: https://www.mdpi.com/article/10.3390/healthcare14142047/s1, Table S1: Distribution of Etiologies of Medical Damage Dispute Cases; Table S2: Distribution of Involved Clinical Departments in Medical Damage Dispute Cases.
