# Supplementary material for: Causes, Characteristics, and Risk Factors of Medical Damage Liability Disputes: A 13-Year Retrospective Analysis of 1761 Litigation Cases in Chongqing, China
Source: Healthcare (Basel). 2026 Jul 8;14(14):2047. doi: 10.3390/healthcare14142047 (PMC13410058; doi:10.3390/healthcare14142047)
Supplement: Supplementary file 1 [file healthcare-14-02047-s001.zip › healthcare-4312376-supplementary.pdf]

Supplementary Table S1. Distribution of Etiologies of Medical Damage Dispute Cases.

Dispute Etiology	Number of Cases (n)	Proportion (%)	Average Actual Compensation ($\bar{x} \pm s$) / 10000 CNY	95% CI (LL, UL)
**Deficiencies in Diagnostic and Therapeutic Procedures**	**887**	50.37%	**22.12 ± 1.02**	(20.1165,24.119)
Improper Treatment Process	350	19.88%	23.10 ± 1.80	(19.5482,26.6462)
Intraoperative Precision Issues	288	16.35%	20.81 ± 1.60	(17.6563,23.9584)
Delayed Treatment Timing	139	7.89%	25.32 ± 2.67	(20.0295,30.602)
Postoperative Complications	52	2.95%	18.06 ± 3.66	(10.7037,25.4129)
Inadequate Preoperative Assessment and Preparation	44	2.50%	19.15 ± 3.86	(11.3757,26.9266)
Excessive Diagnostic and Therapeutic Interventions	14	0.80%	17.24 ± 3.95	(8.6972,25.78)
**Deficiencies in Communication and Informed Consent Management**	**131**	7.44%	**10.00 ± 1.82**	(6.4049,13.5956)
Failure to Fulfill Disclosure Obligations	109	6.19%	11.19 ± 2.13	(6.9707,15.4106)
Ineffective Physician-Patient Communication	22	1.25%	4.10 ± 2.06	(-0.1912,8.3957)
**Failure to Fulfill Duty of Care**	**255**	14.48%	**18.86 ± 1.21**	(16.474,21.2383)

Dispute Etiology	Number of Cases (n)	Proportion (%)	Average Actual Compensation ($\bar{x} \pm s$) / 10000 CNY	95% CI (LL, UL)
Inadequate Patient Condition Monitoring	238	13.52%	19.20 ± 1.27	(16.7041,21.6958)
Insufficient Surveillance	17	0.97%	14.04 ± 3.74	(6.1054,21.9805)
**Diagnostic Deficiencies**	**247**	14.03%	**18.74 ± 1.53**	(15.7298,21.7597)
Missed Diagnosis / Incomplete Diagnostic Coverage	117	6.64%	16.36 ± 1.86	(12.6739,20.0362)
Misdiagnosis	77	4.37%	23.29 ± 3.36	(16.6017,29.9822)
Delayed Diagnosis	33	1.87%	17.70 ± 2.94	(11.7227,23.6815)
Ancillary Examination Errors	20	1.14%	16.94 ± 6.88	(2.5366,31.3394)
**Deficiencies in Nursing Service Management**	**38**	2.16%	**16.42 ± 2.80**	(10.7543,22.0921)
Improper Nursing Procedures Leading to Infection or Complications	14	0.80%	12.30 ± 1.75	(8.5147,16.0795)
Failure to Fulfill Nursing Responsibilities	14	0.80%	24.83 ± 6.91	(9.8956,39.7673)
Infusion / Injection Issues	10	0.57%	10.43 ± 1.34	(7.4002,13.4558)
**Medical Product Issues**	**29**	1.65%	**9.62 ± 2.11**	(5.3007,13.9337)
Improper Use of Medical Products	13	0.74%	10.40 ± 2.37	(5.2428,15.5556)
Infection Resulting from Improper Blood Transfusion	8	0.45%	12.31 ± 6.62	(-3.3501,27.9651)

Dispute Etiology	Number of Cases (n)	Proportion (%)	Average Actual Compensation ($\bar{x} \pm s$) / 10000 CNY	95% CI (LL, UL)
Inherent Quality Defects of Medical Products	8	0.45%	5.66 ± 1.23	(2.7392,8.5733)
**Deficiencies in Medical Documentation Management**	**67**	3.80%	**12.61 ± 2.03**	(8.5535,16.6656)
Non-standard Medical Record Documentation	43	2.44%	8.43 ± 1.68	(5.0402,11.8277)
Falsification of Medical Records	14	0.80%	23.87 ± 6.68	(9.4427,38.3016)
Concealment of Medical Records	6	0.34%	16.46 ± 6.83	(-1.1089,34.0189)
Inadequate Medical History Taking	4	0.23%	12.31 ± 9.17	(-16.8633,41.4833)
**Hospital Management Deficiencies**	**50**	2.84%	**21.62 ± 4.23**	(13.108,30.1288)
Issues Regarding Practice Qualifications and Compliance	35	1.99%	26.19 ± 5.82	(14.3759,38.0109)
Administrative Management Issues	15	0.85%	10.94 ± 2.45	(5.6923,16.1944)
**Improper Medication Use or Adverse Drug Reactions**	**57**	3.24%	**16.75 ± 2.33**	(12.0898,21.4127)
Improper Medication Use	33	1.87%	19.53 ± 3.30	(12.8101,26.2415)
Non-standard Drug Administration	17	0.97%	17.21 ± 3.88	(8.9838,25.4432)

Dispute Etiology	Number of Cases (n)	Proportion (%)	Average Actual Compensation ($\bar{x} \pm s$) / 10000 CNY	95% CI (LL, UL)
Adverse Drug Reaction Events	4	0.23%	3.32 ± 1.29	(-0.7917,7.4367)
Drug Allergy Events	3	0.17%	1.52 ± 1.32	(-4.1702,7.2036)

Note: This table follows a "primary etiology-oriented" classification principle: the primary categories delineate the principal domains of liability, while the secondary entries correspond to specific points of contention. Items possessing independent legal evaluative or preventive significance (e.g., "improper medication use") are listed separately for differentiation. This structure is intended to reflect the authentic compositional characteristics of medical damage incidents.

Supplementary Table S2. Distribution of Involved Clinical Departments in Medical Damage Dispute Cases.

Department	Number of Cases (n)	Proportion (%)	Average Actual Compensation (x̄ ± s) / 10000 CNY	95% CI (LL, UL)
**Internal Medicine**	**214**	12.15%	**20.99 ± 1.57**	(17.901,24.0775)
Rheumatology and Immunology	5	0.28%	21.07 ± 7.94	(-0.9645,43.1085)
Respiratory Medicine	38	2.16%	19.35 ± 3.08	(13.1058,25.5932)
Endocrinology	2	0.11%	11.64 ± 9.91	(-114.2785,137.5585)
Neurology	41	2.33%	23.30 ± 4.76	(13.6751,32.9283)
Nephrology	11	0.62%	23.91 ± 5.11	(12.5198,35.2948)
Gastroenterology	26	1.48%	16.82 ± 2.73	(11.2037,22.4317)
Cardiovascular Medicine	49	2.78%	23.30 ± 3.03	(17.2179,29.3907)
Hematology	7	0.40%	4.05 ± 1.59	(0.1483,7.946)
Oncology	14	0.80%	23.86 ± 10.86	(0.3963,47.3251)
Critical Care Medicine	21	1.19%	22.28 ± 3.64	(14.6785,29.8815)
**Surgery**	**811**	46.05%	**19.26 ± 0.98**	(17.3428,21.1806)
Hepatobiliary Surgery	109	6.19%	26.03 ± 3.72	(18.6548,33.4133)
Anorectal Surgery	8	0.45%	25.65 ± 10.13	(1.6878,49.6072)
Orthopedics	398	22.60%	16.90 ± 1.35	(14.2529,19.5452)
Urology	69	3.92%	19.77 ± 2.79	(14.2028,25.3276)
Breast and Thyroid Surgery	16	0.91%	21.17 ± 2.74	(15.332,27.0068)
Neurosurgery	48	2.73%	23.82 ± 4.39	(14.987,32.643)
Gastrointestinal	85	4.83%	19.86 ± 2.77	(14.3582,25.3644)

Department	Number of Cases (n)	Proportion (%)	Average Actual Compensation ($\bar{x} \pm s$) / 10000 CNY	95% CI (LL, UL)
Surgery				
Cardiothoracic Surgery	43	2.44%	22.06 ± 3.49	(15.021,29.0925)
Vascular Surgery	13	0.74%	13.86 ± 2.70	(7.9839,19.7392)
Medical Aesthetics / Cosmetic Surgery	22	1.25%	8.64 ± 1.68	(5.1417,12.1365)
**Pediatrics**	**99**	5.62%	**26.07 ± 3.36**	**(19.404,32.7342)**
General Pediatrics	65	3.69%	25.73 ± 4.30	(17.1441,34.307)
Neonatology	34	1.93%	26.73 ± 5.40	(15.7491,37.7027)
**Obstetrics and Gynecology**	**307**	17.43%	**22.04 ± 1.56**	**(18.9783,25.1016)**
Obstetrics	244	13.86%	23.92 ± 1.88	(20.2044,27.6304)
Gynecology	63	3.58%	14.77 ± 1.79	(11.1848,18.3523)
**Specialty Medicine**	**142**	8.06%	**14.20 ± 2.11**	**(10.0228,18.3801)**
Otorhinolaryngology (ENT)	24	1.36%	30.48 ± 9.40	(11.0334,49.9191)
Infectious Diseases	3	0.17%	2.00 ± 1.04	(-2.4854,6.492)
Psychiatry	24	1.36%	20.48 ± 4.01	(12.1861,28.7647)
Rehabilitation Medicine	14	0.80%	7.87 ± 2.13	(3.2596,12.479)
Stomatology / Oral Medicine	24	1.36%	13.53 ± 5.58	(1.9828,25.078)
Dermatology	10	0.57%	9.14 ± 3.12	(2.0719,16.2041)
Ophthalmology	39	2.21%	5.22 ± 0.83	(3.5445,6.9052)
Preventive Healthcare	4	0.23%	14.43 ± 3.81	(2.3047,26.5503)

Department	Number of Cases (n)	Proportion (%)	Average Actual Compensation ($\bar{x} \pm s$) / 10000 CNY	95% CI (LL, UL)
**General Practice**	**74**	4.20%	**11.08 ± 1.75**	**(7.5921,14.5644)**
General Practice	74	6.47%	11.08 ± 1.75	(7.5921,14.5644)
**Emergency Medicine**	**114**	12.15%	**16.26 ± 1.53**	**(13.2386,19.2851)**
Emergency Medicine	114	0.28%	16.26 ± 1.53	(13.2386,19.2851)

Note: This table adopts a hybrid classification system based on clinical operational units: primary labels represent broad administrative categories (e.g., Internal Medicine), while secondary labels denote specific subspecialties (e.g., Cardiovascular Medicine). Given the substantial variation in risk profiles across subspecialties, the subspecialty level is retained for independent presentation to prevent information aggregation from obscuring risk characteristics. Primary categories without listed subspecialties (e.g., Emergency Medicine) are treated as independent analytical units. This classification is intended to balance hierarchical clarity with precision in risk analysis.
